# Supplementary material for: Complexome profiling of the Chlamydomonas psb28 mutant reveals TEF5 as an early PSII assembly factor
Source: Plant Cell. 2025 Mar 17;37(6):koaf055. doi: 10.1093/plcell/koaf055 (PMC12164590; doi:10.1093/plcell/koaf055)
Supplement: koaf055_Supplementary_Data [file koaf055_supplementary_data.zip › psb28_interactive.zip!/plots/intensity/qualityPlots/peptides/_Cre12.g515500.t1.1.html]

### Cre12.g515500.t1.1

Trivial name:   
SeqCoverage [median]: 0.60  
Mass: 345.42 kDa  
  
 

| Sequence | MissedCleavage | Mass [Da] | wtIntensity | muIntensity | Score |
| --- | --- | --- | --- | --- | --- |
| ELAKQAEEEAR | 1 | 1272.63 | 1010.63 | 0.00 | 39.92 |
| RELEELEMQR | 1 | 1331.65 | 1023.77 | 6272.03 | 50.29 |
|  |  |  | 2034.4 | 6272.0 |  |
